# Fluorescence Photobleaching as an Intrinsic Tool to Quantify the 3D Expansion Factor of Biological Samples in Expansion Microscopy

**DOI:** 10.1021/acsomega.0c00118

**Published:** 2020-03-17

**Authors:** Marisa Vanheusden, Raffaele Vitale, Rafael Camacho, Kris P. F. Janssen, Aline Acke, Susana Rocha, Johan Hofkens

**Affiliations:** Department of Chemistry, KU Leuven, Leuven 3000, Belgium

## Abstract

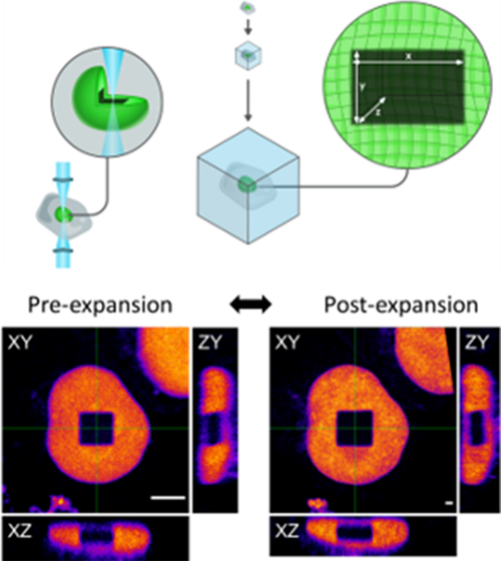

Four years after its first report, expansion microscopy (ExM) is now being routinely applied in laboratories worldwide to achieve super-resolution imaging on conventional fluorescence microscopes. By chemically anchoring all molecules of interest to the polymer meshwork of an expandable hydrogel, their physical distance is increased by a factor of ∼4–5× upon dialysis in water, resulting in an imprint of the original sample with a lateral resolution up to 50–70 nm. To ensure a correct representation of the original spatial distribution of the molecules, it is crucial to confirm that the expansion is isotropic, preferentially in all three dimensions. To address this, we present an approach to evaluate the local expansion factor within a biological sample and in all three dimensions. We use photobleaching to introduce well-defined three-dimensional (3D) features in the cell and, by comparing the size and shape pre- and postexpansion, these features can be used as an intrinsic ruler. In addition, our method is capable of pointing out sample distortions and can be used as a quality control tool for expansion microscopy experiments in biological samples.

## Introduction

Fluorescence microscopy has proven to be a very valuable tool for accurately unraveling fundamental biological questions by optically magnifying the image of specifically stained structures of interest.^1^ However, in biology, the biomolecular actors of many crucial cellular processes often require imaging with a nanoscale resolution. As the resolution of a conventional fluorescence microscope is inherently limited by the physical laws governing the diffraction of light,^2^ correctly locating these biomolecular actors is challenging. To lift this barrier, different microscopy concepts, known under the term “super-resolution fluorescence microscopy,” have been developed independently.^3−8^ Nonetheless, these methods typically require complex microscopy setups or special fluorescent labels. For this reason, Boyden and co-workers introduced expansion microscopy (ExM) as an alternative to classical super-resolution (SR) fluorescence microscopy techniques for obtaining sub-diffraction resolution on conventional fluorescent microscopes and with classic fluorescent labels.^9^ Rather than by increasing the optical resolution, the physical distance between independent fluorophores is increased by expanding the sample inside a swellable polymer. To achieve this, structures of interest such as proteins (Pro-ExM)^10^ or nucleic acids^11^ require to be labeled with an additional chemical cross-linker, besides the classic fluorescent labeling in order to ensure downstream covalent grafting of their original location in the hydrogel. Alternatively, recently, multifunctional linkers have been introduced containing a biomolecular probe, a fluorescent reporter, and a cross-linkable group, leading to better preserved fluorescent signals and a broader scope of biological targets.^12,13^ Finally, by infusing the samples with monomers, a hydrogel can be formed within the prestained sample. Physical expansion is achieved upon dialysis in water, with an expansion factor (EF) of 4–10×, which depends on the type of hydrogel that is being used, resulting in a resolution of up to 25 nm on conventional microscopes.^14^

Its ease of implementation and the inherent clearing of the sample has led to the rapid adoption of ExM in the field of fluorescence microscopy.^10−19^ Not only has this led to the successful imaging of different tissue slices^9,16^ in combination with different super-resolution approaches but ExM also seems suitable for uncovering the structure of different protein complexes given that the expanded imprint of the sample perfectly mimics its original conformation.^20−22^ However, it currently remains a challenge to objectively evaluate whether this assumption holds in different biological samples. That is to say, it remains unclear whether the expansion process is isotropic in three dimensions so that the relative orientation of the sample components remains unchanged. Tinnefeld and co-workers have used DNA nanorulers to evaluate the expansion factor of the polymer network on the relevant nano- and microscales.^23^ They reported a microscopic expansion factor of ∼3× with a homogeneous distribution throughout the sample. However, this finding proved that the isotropic expansion of the polymer network itself does not yet completely excludes the introduction of distortions in an actual biological sample. As previously reported by Tillberg et al., incomplete homogenization of the sample’s mechanical characteristics could alter the behavior of the hydrogel.^24^ Furthermore, the use of DNA nanorulers only enabled the quantification of the expansion process in the *x*- and *y*-dimension and only close to the glass interface, where, as suggested by the authors themselves, the expansion factor might be reduced due to frictional forces. Therefore, several other methods are currently being used to calculate the expansion factor and evaluate the isotropic expansion in an actual biological sample. Originally, Boyden et al. evaluated their expansion process by comparing the post-ExM image to the pre-ExM image. However, the expansion process is hereby only quantified in the *xy*-plane. Due to optical sectioning with a confocal microscope and due to the sample expansion in the *z*-dimension, it is impossible to acquire the same features in the pre- and the postexpansion images. As such, the images cannot always be successfully compared. Other ways to quantify the expansion process of a biological sample, e.g., by making use of a nuclear pore complex,^25^ again require the application of additional super-resolution imaging approaches such as STED^18,20^ or SIM,^22^ revoking the purpose of using ExM as an alternative for super-resolution (SR) microscopy. For these reasons and considering the heterogeneous distribution of the expansion factor (EF) in the hands of different scientists, verifying the isotropy of the expansion process of biological samples remains a key challenge.

**Figure 1:**
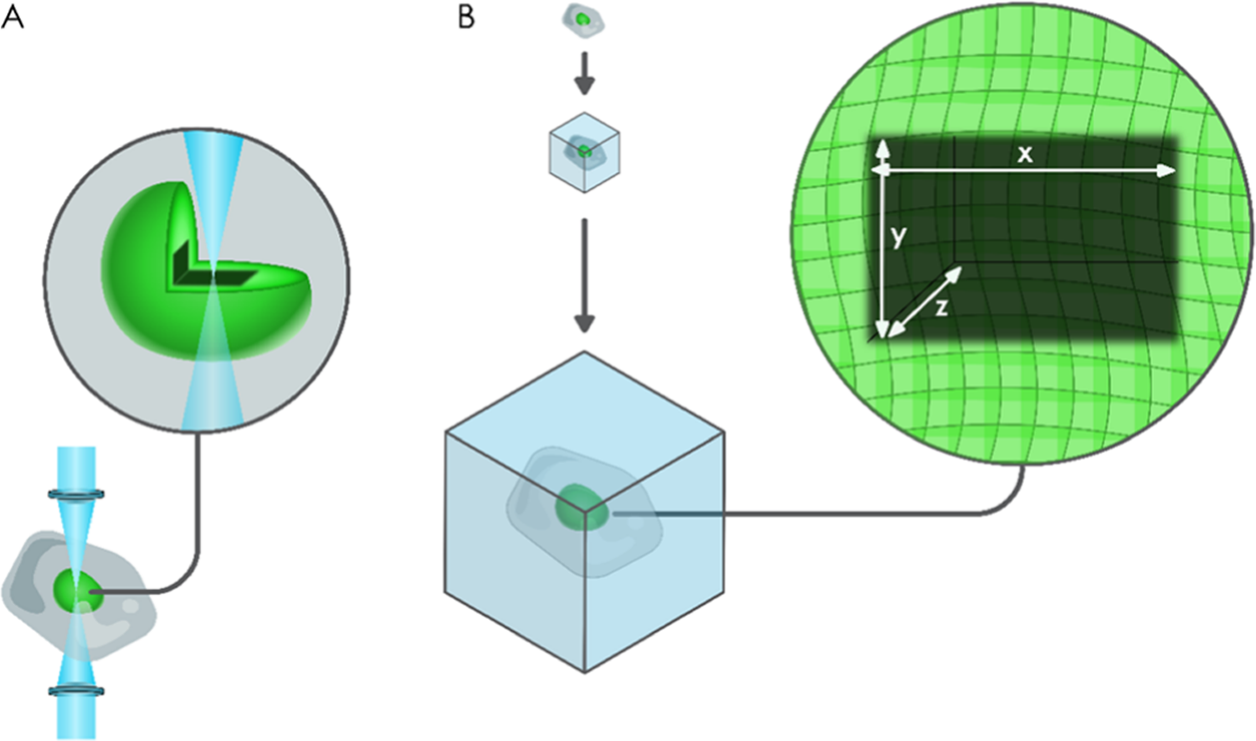
Schematic representation of the photobleaching experiment for the evaluation of the expansion process. (A) Using photobleaching, a well-defined cube is bleached in an EGFP-stained nucleus. (B) Photobleached samples are then embedded in the polymer matrix, expanded, and imaged in 3D by a confocal microscope.

Here, we propose a concept that addresses the quantification of the expansion process in 3D, within biological samples and without the need for other super-resolution approaches. Through fluorescence photobleaching, the photochemical process by which fluorochromes permanently lose the ability to fluoresce due to photon-induced modification,^26^ we created an intrinsic ruler under the form of a well-defined cube inside the cell’s nucleus (Figure 1). Subsequent comparison of these structures with the postexpansion image easily reveals the isotropic nature of expansion microscopy in both lateral and axial dimensions. Introducing a bleached cubical feature within a biological sample has several advantages over the comparison of pre- and postexpansion images. First, the bleached cube provides the sample with a regular geometrical shape, instead of more random features, thus rendering the subsequent data analysis an easier, more immediate, and, possibly, more robust task. Furthermore, photobleaching emphasizes pixel intensity differences, dramatically simplifying image segmentation. As a consequence of the previous point, photobleaching permits to easily identify the same sample area pre- and postexpansion, which can otherwise be a challenging task due to similarities of patterns present in the sample. Although we use two-photon excitation (2PE) to assess the isotropy in 3D, our method operates on two-dimensional projections of 3D image stacks; therefore, it is directly applicable to the data resulting from both one- and two-photon excitation experiments.

## Results and Discussion

### Creating Well-Defined Bleaching Patterns in 3D

**Figure 2:**
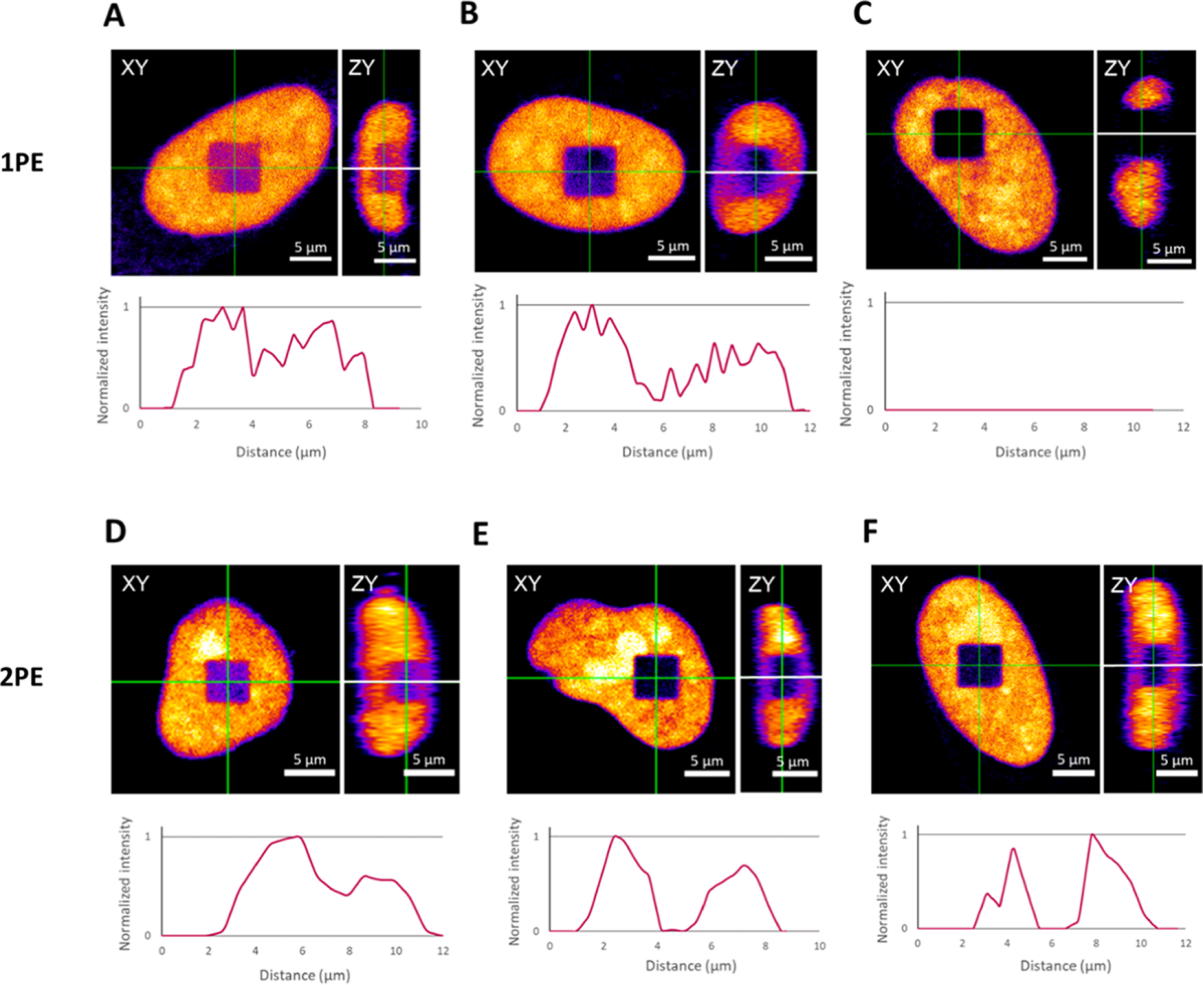
Empirically determined optimal bleaching conditions for one-photon (1PE) and two-photon (2PE) excitations. For each condition, both the *xy*-plane and the orthogonal view of the *zy*-plane are shown. Additionally, normalized intensity profiles registered along the *z*-direction (white solid lines) are included. Upper panel: 1PE with a laser power of 8.7 μW, a pixel dwell time of 2.09 μs, and a repetition of (A) 3, (B) 10, and (C) 20 frames. Lower panel: 2PE with (D) a laser power of 3.9 mW, a pixel dwell time of 2.09 μs, and a repetition of seven frames and (E) a laser power of 6.3 mW, a pixel dwell time of 2.09 μs, and a repetition of five frames or (F) a repetition of four frames. Pixel sizes varied depending on the area selected. All laser powers were measured at the objective. Scale bars: 5 μm.

To obtain a well-defined 3D photobleached structure in a biological sample, we selected the cell’s nucleus as a target of choice due to its micrometer scale, presence in all biological samples, and suitability for uniform labeling (see below). Preferentially, the resulting photobleached cube has a high contrast along *x*-, *y*-, and *z*-axis and the edges of its structure fit well within the boundaries of the cell’s nucleus. Although we have explored the commonly used nuclear 4′,6-diamidino-2-phenylindole (DAPI) staining for the introduction of photobleached cubes, we noticed a partial recovery of the fluorescent signal in the postexpansion image, likely due to the inability to cross-link the DAPI molecule to the polymer network (Figure S3). Eventually, for demonstrating the full potential of the analysis methodology, we selected the enhanced green fluorescent protein (eGFP). Besides the fact that eGFP can be directly cross-linked to the polymer matrix, fluorescent proteins such as GFP are the labels of choice in other photobleaching-based assays such as in fluorescence recovery after photobleaching (FRAP) as they are known to bleach quite easily and irreversibly while being rather stable under low-intensity imaging conditions, which is desirable for postexpansion imaging.^27^ The rate at which GFP bleaches can be fine-tuned and varies among different GFP mutants.^28−30^ As such, we first transfected HeLa cells with a vector for nuclear eGFP expression to generate photobleached cubic structures inside the nucleus. While a uniformly distributed signal was observed, the nucleus of the cells was relatively flattened. Hence, the height of the cell’s nucleus was not sufficient to fit the typical *z*-dimension of a bleached cube. Next, we tested different photobleaching conditions in a HeLa cell line stably expressing histone2B-eGFP. The expression of the histone2B-eGFP indeed gives more volume to the nucleus. However, here we found that due to the inhomogeneous distribution of the eGFP signal throughout the nucleus, squares showed irregular shapes. Finally, we selected the histone2B-eGFP expressing cells, further transfected with the vector for nuclear eGFP expression, as our target of choice. We empirically determined the optimal bleaching conditions for this target, starting with one-photon excitation (1PE). While standard confocal microscopy with 1PE utilizes a pinhole to exclude out-of-focus light originating from the emission, the excitation light does excite and thus photobleaches fluorophores throughout the specimen. While the borders of the cube are well-defined in the *xy*-dimension, 1PE does not allow to acquire well-defined 3D structures in the *z*-dimension, which fit our requirements. In more detail, bleaching conditions used in Figure 2A were not sufficient to provide a high enough contrast between the bleached and nonbleached areas, especially in the *z*-dimension, as can be concluded from the normalized intensity profile. While repetitive scanning did improve the contrast in each dimension, the edges of the cube in the *z*-dimension were still not well-defined (Figure 2B). Further increasing the number of repeated scanning steps resulted in cubes that completely exceed the boundaries of the nucleus (Figure 2C), making downstream segmentation in the axial direction difficult or impossible. This can be concluded from both the fluorescence images and the corresponding normalized intensity profiles along the *z*-dimension. To exclude the impact of photobleaching introduced in out-of-focus planes on the dimensions of our photobleached cube, we performed a similar experiment where we tested different bleaching parameters with 2PE. The concept of 2PE employs the absorption of two photons, each carrying approximately half the energy needed for excitation in a single quantum event. As the probability for this nearly simultaneous absorption of two photons is only high enough in the focal volume, the spread of the excitation volume along the *z*-dimension is substantially lower in 2PE, reducing the photobleaching of emitters outside the focal plane.^31^ Here, the laser power used to generate the image in Figure 2D was insufficient to result in a highly contrasted cube, even after seven repetitive scans. By increasing the laser power but reducing the frame repetition to four or five times (Figure 2E,F respectively), better contrast was observed. Thus, as expected for 2PE, we did notice an overall more confined structure when looking at the *z* cross section of the cube. We now aim at demonstrating the utility of photobleaching to extract the EF in both lateral and axial dimensions.

### Evaluation of the Expansion Process in 3D

**Figure 3:**
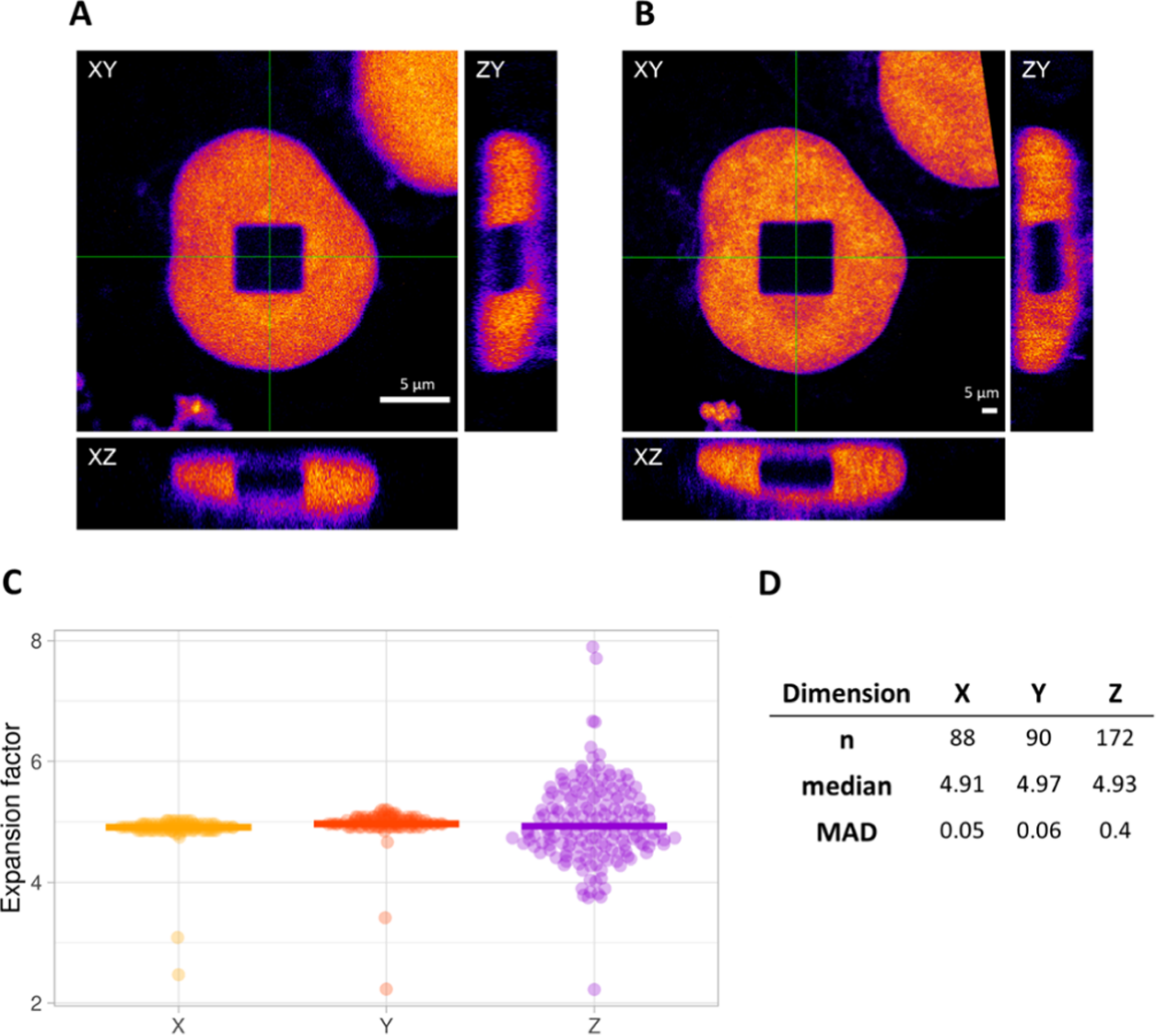
Example of an isotropic cube. Orthogonal views (A) pre- and (B) post-expansion. Scale bars: 5 μm. (C) Distribution of the expansion factors calculated for each corresponding pixel line in the *x*-, *y*-, and *z*-dimensions for the example shown in (A) and (B). Lines represent median values. In *z*-dimension, we clearly see a broader distribution for the expansion factor, as the resolution in this dimension is typically lower. Single outliers that originate from the pixels at the borders of the square that were below 2 and above 10 were removed, as they are not representative of the actual expansion factor. Outliers that originated from pixel lines in the middle of the square were never removed. (D) Size, median value, and median absolute deviation (MAD) of the sample distributions plotted in (C).

Using the optimal photobleaching conditions (Figure 2E,F) for the generation of the cubes in the cell’s nucleus, we first quantified the expansion factor. After photobleaching, *z*-stacks of the photobleached cube were acquired both pre- and postexpansion. Next, the expansion factor can be easily extracted by dividing the dimensions of the cube after expansion by the dimensions pre-expansion. As such, for the cell in Figure 3, we obtained an expansion factor of 4.9×, which is 0.4× higher than the expansion factor reported in the first example of ExM.^9^ However, expansion factors may vary due to small differences in the composition of the monomer solution or due to the efficiency of sample homogenization.

**Figure 4:**
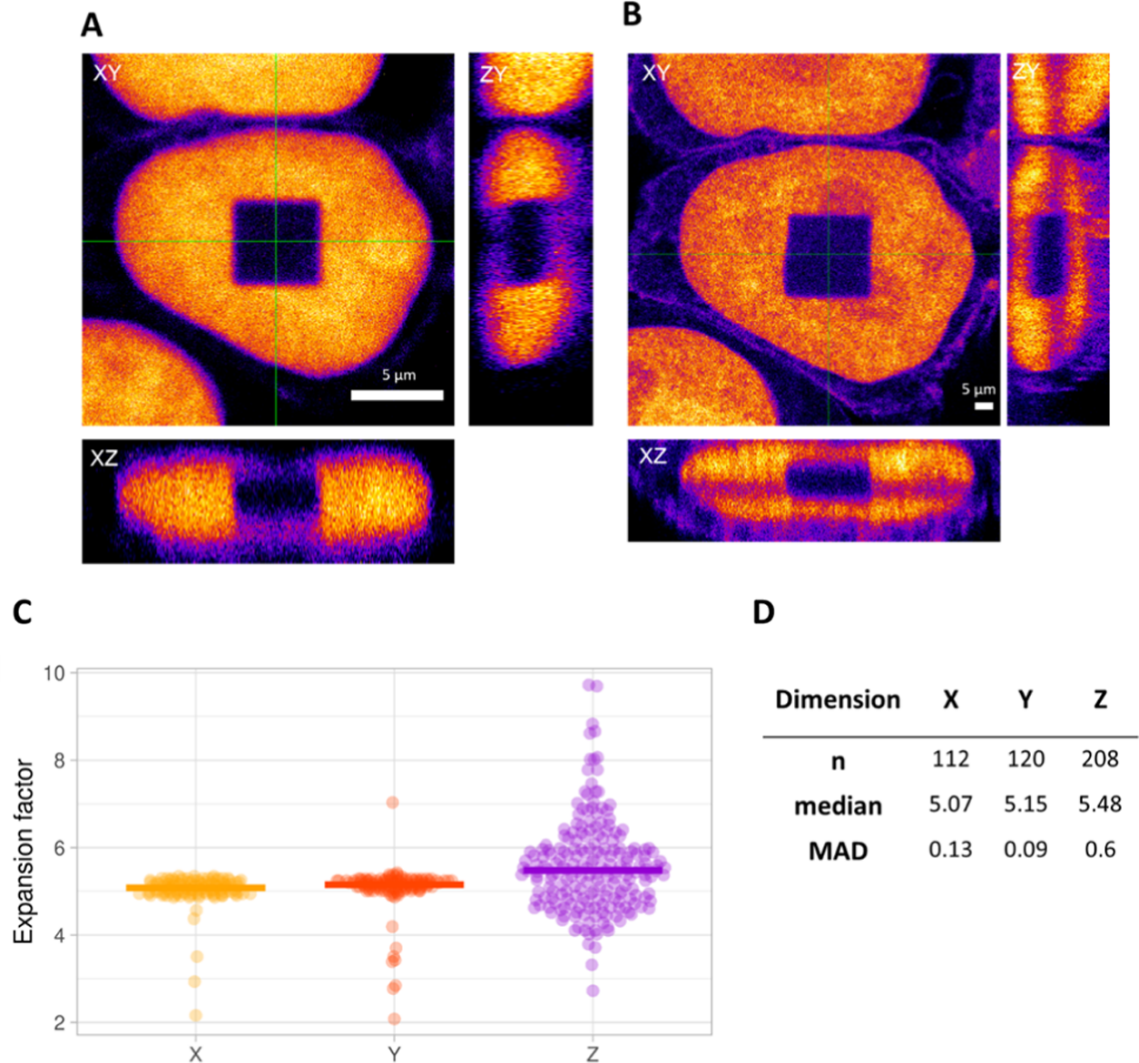
Example of a cube with a local distortion. Orthogonal views (A) pre- and (B) postexpansion. Scale bars: 5 μm. (C) Distribution of the expansion factors calculated for each corresponding pixel line in the *x*-, *y*-, and *z*-dimension for the example shown in (A) and (B). Lines represent median values. In *z*-dimension, we clearly see a broader distribution for the expansion factor, as the resolution in this dimension is typically lower. Single outliers that originate from the pixels at the borders of the square that were below 2 and above 10 were removed, as they are not representative of the actual expansion factor. Outliers that originated from pixel lines in the middle of the square were never removed. (D) Size, median value, and median absolute deviation (MAD) of the sample distributions plotted in (C).

Second, the expansion process was evaluated in three dimensions. Even though the photobleaching approach enables an assessment of the expansion process by eye, we demonstrate how to trace potentially introduced distortions in the postexpanded sample using a quantitative approach. By comparing the actual length of each individual pixel line in the pre-expansion image to its corresponding pixel line in the postexpansion image, local expansion factors can be obtained. To make sure that we compare the same planes, we first define the image that displays the middle plane of the cube for each dimension. The resulting local expansion factors were plotted together with their median value in a violin plot. We opted for the median value, as it is less influenced by outliers. We noted that outliers are expected for each data set, as the segmented square always shows a few rows with a small number of pixels (Figure S1). However, outliers that originated from the first three lines containing few pixels were excluded from the plot for better visualization. In the first example, we show a cube where the median of the expansion factors for each dimension is almost identical (Figure 3). Furthermore, distributions are very narrow for the *x*- and *y*-dimensions, with a median absolute deviation (MAD) value of less than 0.1×. The MAD value represents the median of the absolute deviation of the different data points from the data’s median. The distribution of expansion factors in the *z*-dimension is typically wider, with a MAD value of 0.4×, which we expected due to the intrinsic lower resolution of the microscope in the axial direction. To report possible local distortions, which have no direct effect on the median values or the MAD value, we introduce another parameter. This parameter is represented by the slope of the plot of the number of pixels along the *x*-dimension as a function of the pixel line (Figure S2). As a photobleached square is intrinsically always straight, the slope of an isotropically expanded square should approach zero. Local anisotropies and distortions, however, cause the slope to deviate from zero. Based on this additional parameter and considering the discussion about the median values and the MAD, we classified the square in Figure 3 as isotropic (Figure S2A). A second example shows a square that displays a local distortion along the *x*-axis (Figure 4). While the median values are slightly more deviating from each other and the MAD is slightly higher for each dimension, the clearest effect of the local distortion is represented in the slope of the plot in Figure S2B. Additionally, as shown in Table S1, isotropy along the *z*-axis has the potential to be concomitant with isotropy along *x*- and *y*-axis. We noticed this trend for a limited number of samples (three additional cells measured; Figure S4 and corresponding Table S1). These findings suggest that even when quantification in 2D is performed, our method is suitable for accurately assessing the general quality of the expansion procedure.

**Figure 5:**
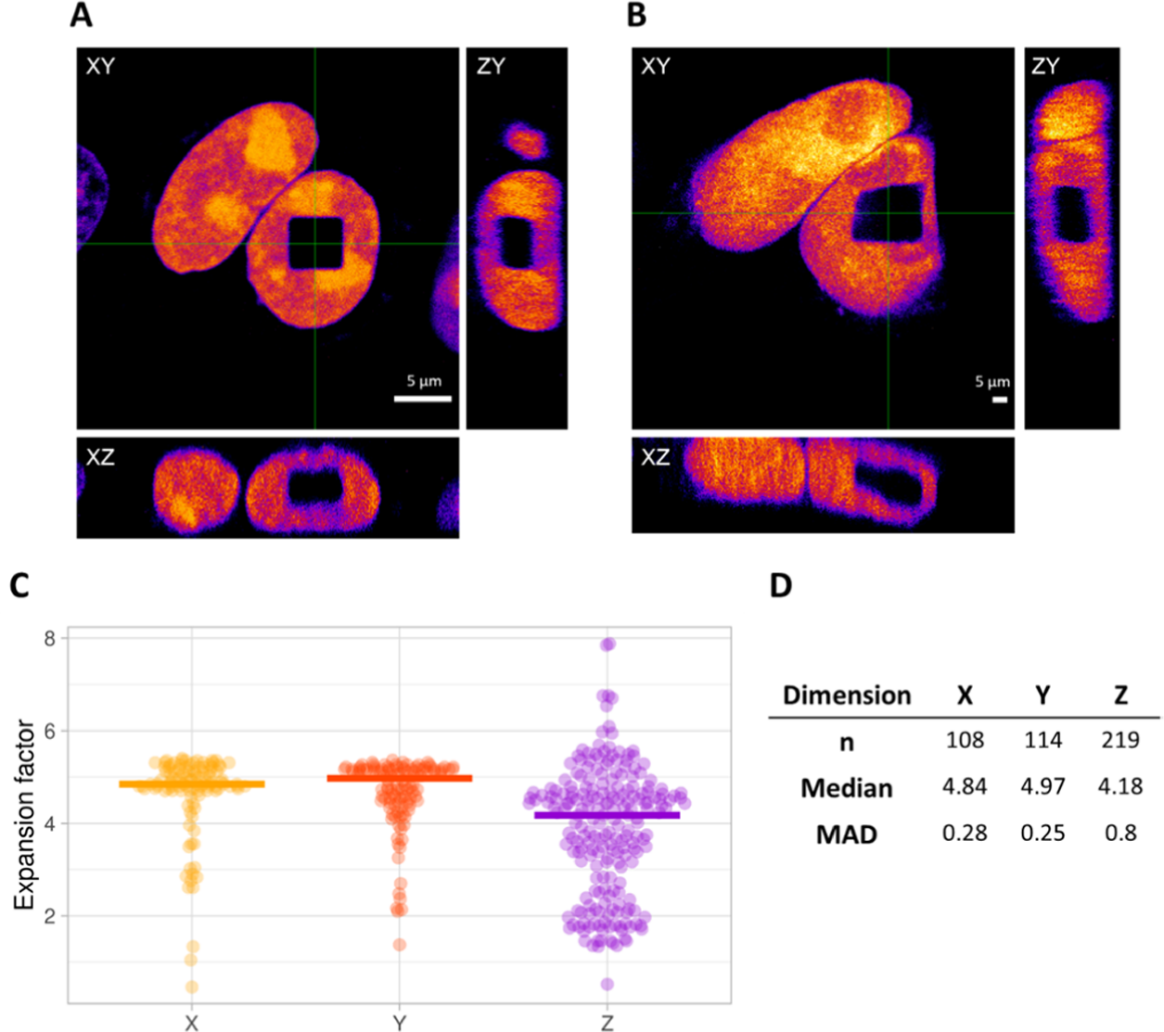
Example of an anisotropic cube. Orthogonal views (A) pre- and (B) postexpansion. Scale bars: 5 μm. (C) Distribution of the expansion factors calculated for each corresponding pixel line in the *x*-, *y-*, and *z*-dimension for the example shown in (A) and (B). Lines represent median values. In *z*-dimension, we clearly see a broader distribution for the expansion factor, as the resolution in this dimension is typically lower. Single outliers that originate from the pixels at the borders of the square that were below 2 and above 10 were removed, as they are not representative of the actual expansion factor. Outliers that originated from pixel lines in the middle of the square were never removed. (D) Size, median value, and median absolute deviation of the sample distributions plotted in (C).

Finally, squares that were classified as anisotropic showed an odd distribution of the expansion factor in the corresponding dimension (Figure 5C). In the *x*- and *y*-dimension, distributions are not nicely confined and the values below the median span a rather wide range of expansion factors. This is expected, as the expansion factors originating from the first and last rows of pixels in the postexpansion image are rather short due to distortions (Figure 5B). When compared to their corresponding pixel lines in the original square, this will result in low expansion factors. Furthermore, the distribution for the *z*-dimension clearly shows two regimes, *i*.*e*., the first group of expansion factors is centered around 5× and a second around 2×. This result originates from the distortion observed on the *xz*-plane (Figure 5B), where the left part of the square appears less expanded than the right part.

## Conclusions

Previously reported approaches to quantify the expansion process in fluorescence expansion microscopy did not address this task in 3D or need to be completed by super-resolution techniques. To overcome these limitations, we developed a new method that uses photobleaching to permanently mark the sample with a well-defined 3D cube, which could then serve as an intrinsic reporter for validating isotropic expansion both in lateral and axial dimensions. We, however, noticed that for our single-cell experiments, 2PE was needed to obtain a nice contrast between the bleached square and sample in the axial dimension. Nevertheless, for laboratories with exclusive access to a confocal microscope, this strategy also results in a more straightforward way to evaluate the quality of the expansion process in 2D, as a confocal setup is perfectly capable of introducing nicely confined structures in the *xy*-plane. Although we decided to evaluate the potential of this new methodology using the cell nucleus with the overexpression of EGFP, the robustness of the developed data analysis approach allows different labeling strategies to be explored depending on the specific sample requirements. With the proposed methodology, we found that the expansion process intrinsically is isotropic in 3D at the length scales investigated, with an expansion factor of ∼5×. However, we do note that the risk of generating distortions during the expansion process remains a potential limitation of the technique. We noticed that some of our samples displayed irregularities. As the manual transfer of the expanded gels to a poly-lysine-coated coverslip (for stabilization purposes) is the most critical experimental stage of our laboratory protocol, we hypothesize that the aforementioned distortions are somehow induced during the gel transfer stage. If this could be confirmed in the future, one can develop modified handling protocols; e.g., one could think of stabilizing the gel during imaging without transferring it. Regardless, the developed procedure enables both their fast visual evaluation and more robust quantification of the local expansion factors.

## Materials and Methods

### Cell Culture and Transfection

HeLa histone eGFP cells were obtained from ATCC and cultured in Dulbecco’s modified Eagle’s medium (DMEM) without phenol-red (Gibco Life Technologies, Invitrogen) supplemented with 10% fetal bovine serum (FBS), 1% glutamax, and 50 μg/mL gentamicin at 37 °C with 5% CO_2_. Twenty-four hours before transfection, 2.4 × 10^5^ cells suspended in 3 mL of complete growth medium were seeded in a 35 mm glass-bottom dish (ThermoFisher Scientific) equipped with an adhesive silicone isolator (24-well, Grace Bio-Labs) with 4.5 mm feature well diameter. The pEGFP plasmid was a kind gift from Prof. Hideaki Mizuno. The vector (50 ng/well) was transfected into cells with 0.1 μL/well of TransIT-X2 (Mirus Bio) according to the manufacturer’s instructions. Sixteen hours after transfection, the cells were washed once with prewarmed phosphate-buffered saline (PBS) and then fixed with 4% paraformaldehyde in PBS for 15 min at room temperature. After fixation, the cells were washed twice with PBS, permeabilized for 15 min using PBS containing 0.1% Triton X-100, and washed three times for 5 min using PBS before proceeding to photobleaching.

### Photobleaching

All photobleaching experiments reported in Figures 3–5 were performed with two-photon excitation using a Leica TCS SP8 X system (Leica Microsystems) equipped with a Mai Tai DeepSee laser (Spectra-Physics) and a 63× NA 1.2 objective lens. The desired region, defined as a regular *xy*-plane, was exposed to a laser power of 6.3 mW at 968 nm, with a pixel dwell time of 2.09 μs per pixel. Each frame was scanned four times under the above-mentioned conditions. The focal volume was positioned more or less at the center of the *z* cross section of the nucleus to prevent the bleached area from overlapping the edges of the nucleus and causing the corners of the square to be undefined. Squares were found to typically have an axial height of 5–10 μm. For imaging the nucleus containing the bleached volume, a *z*-stack series was acquired using the confocal setup and white light laser at 488 nm and following the Nyquist criterion for the determination of both the *z*-step size and the pixel size.

### Gelation, Digestion, and Expansion

Sample expansion was performed as described previously. In brief, the cells were incubated for 12 h in 0.1 mg/mL Acryloyl-X, SE (ThermoFisher Scientific) at room temperature and washed two times for 15 min with PBS. Monomer solution (1× PBS, 2 M NaCl, 8.625% w/w sodium acrylate, 2.5% w/w acrylamide, and 0.15% w/w *N*,*N*′-methylenebisacrylamide) was prepared, frozen in aliquots, and thawed before use. To perform the gelation step, the monomer solution was enriched with 0.15% tetramethylenediamine (TEMED) and 0.15% ammonium persulfate (APS) at 4 °C to prevent premature gelation, and 20 μL of this solution was added to each silicone isolator well to embed the cells. Gelation took place at 37 °C for 1 h. Next, the silicone isolator wells were carefully removed and the gels were incubated in 2–3 mL of proteinase K (New England Biolabs) diluted to 8 U/mL in digestion buffer (50 mM Tris, pH 8, 1 mM ethylenediaminetetraacetic acid (EDTA), 0.5% Triton X-100, 0.8 M guanidine HCl) for 12 h at room temperature. Digested gels were next immersed in Milli-Q water, which was exchanged every hour for five times to ensure complete expansion. Upon reaching a plateau of the expansion process, the gels were transferred to a six-well glass bottom plate, coated with poly-d-lysine. The gels were transferred by making use of a 60 × 24 mm^2^ #1.5 coverslip and a paintbrush.

### Fluorescence Microscopy

After completing cross-linking, polymerization, homogenization, and expansion, the bleached cubes were retrieved in the expanded gel using a low-magnification objective first. The postexpansion image acquisition was performed by the same Leica TCS SP8 X confocal system equipped with a 20× NA 1.20 objective lens. The expanded samples were immersed in Milli-Q water to keep the gels in their fully expanded state during image acquisition. Each nucleus containing a bleached cube was imaged in 3D using a white-light laser set at 488 nm for excitation, with 1 Airy unit and Nyquist sampling for both the *z*-step size and the pixel size.

### Data Analysis

To compare the actual dimensions of the cube pre- and postexpansion, stacks of the pre- and postexpanded samples were preprocessed using Fiji. In detail, the postexpansion stack was rotated to match the angle of the pre-expansion stack, and the *xy*-plane lying in the middle of the cube’s height (*i*.*e*., the *z*-dimension) was used for analyzing the distribution of expansion factors along *x*- and *y*-dimension. Next, as the histogram of the intensities of the postexpansion images is typically less spread out, it was rescaled. After this, pre- and postexpansion images were segmented using an intensity-based threshold; a median filter was applied to smoothen the images without perturbing the features’ edges. The squares present in the selected images were selected as a region of interest (ROI), and downstream analysis was run on such a selection to extract the distribution of the expansion factors in the *x*- and *y*-dimension. This was achieved by first matching the dimensions of the pre- and postexpansion squares such that each individual pixel line could be measured and compared. Afterward, the number of black pixels for each pixel line both along *x*- and *y*-dimension was counted, the number of black pixels was multiplied by the pixel size, and the actual dimension (in microns) of each pixel line in the postexpansion image was divided by the actual length of the corresponding pixel line in the pre-expansion image to obtain the expansion factor. Finally, all expansion factors for each dimension were plotted in a violin plot using https://huygens.science.uva.nl/PlotsOfData/. For the *z*-dimension, stacks were resliced in Fiji such that they displayed images of the *xz*- or *yz*-plane as a function of the *y*- or *x*-dimension, respectively; afterward, the stacks were treated exactly the same way as described above.

## Supporting Information Available

Orthogonal views of the thresholded cube; the number of internal pixels along *x*-dimension as a function of the pixel line; photobleaching in a nuclear DAPI staining; cubes that were classified as being isotropic in 3D using the presented approach; and script for Fiji for segmentation and expansion factor calculation (PDF)
