# Supplementary material for: Fluorescence Photobleaching as an Intrinsic Tool to Quantify the 3D Expansion Factor of Biological Samples in Expansion Microscopy
Source: ACS Omega. 2020 Mar 17;5(12):6792–9. doi: 10.1021/acsomega.0c00118 (PMC7114699; doi:10.1021/acsomega.0c00118)
Supplement: Supplementary file 1 — ao0c00118_si_001.pdf [file ao0c00118_si_001.pdf]

Supporting Information

Fluorescence photobleaching as an intrinsic tool to quantify the 3D expansion factor of biological samples in expansion microscopy

Marisa Vanheusden, Raffaele Vitale, Rafael Camacho,[†] Kris P.F. Janssen,[‡] Aline Acke, Susana Rocha,* Johan Hofkens*

Department of Chemistry, KU Leuven, Leuven, Belgium

AUTHOR INFORMATION

* e-mail correspondence to : susana.rocha@kuleuven.be
johan.hofkens@kuleuven.be

Table of Contents

Figure S1: Orthogonal views of thresholded cube..**S3**

Figure S2: Number of internal pixels along *x* as a function of the pixel line **S3**

Figure S3: Photobleaching in a nuclear DAPI staining **S4**

Table S1: Cubes that were classified as being isotropic in 3D using the presented approach. **S4**

Figure S4: Cubes that were classified as being isotropic in 3D using the presented approach....**S5**

Script used in Fiji for segmentation and expansion factor calculation **S6**

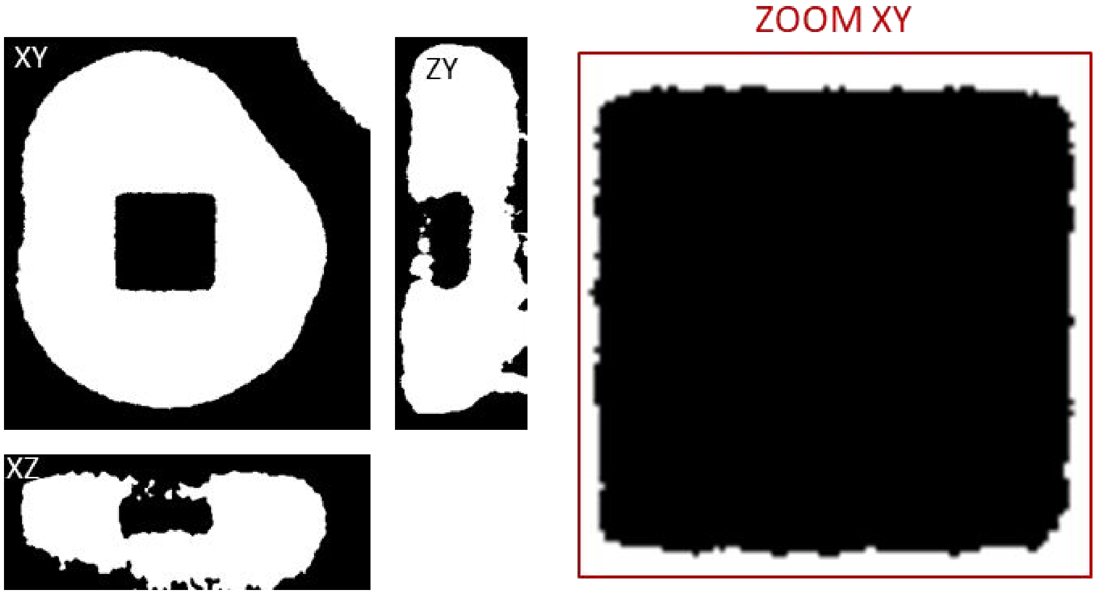

Figure S1: Thresholded version of the cube represented in figure 3. Edges of the cube typically display pixel lines with only a few black pixels, which results in outliers in the distribution of the local expansion factor (excluded from the subsequent data processing for the sake of a better visualization).

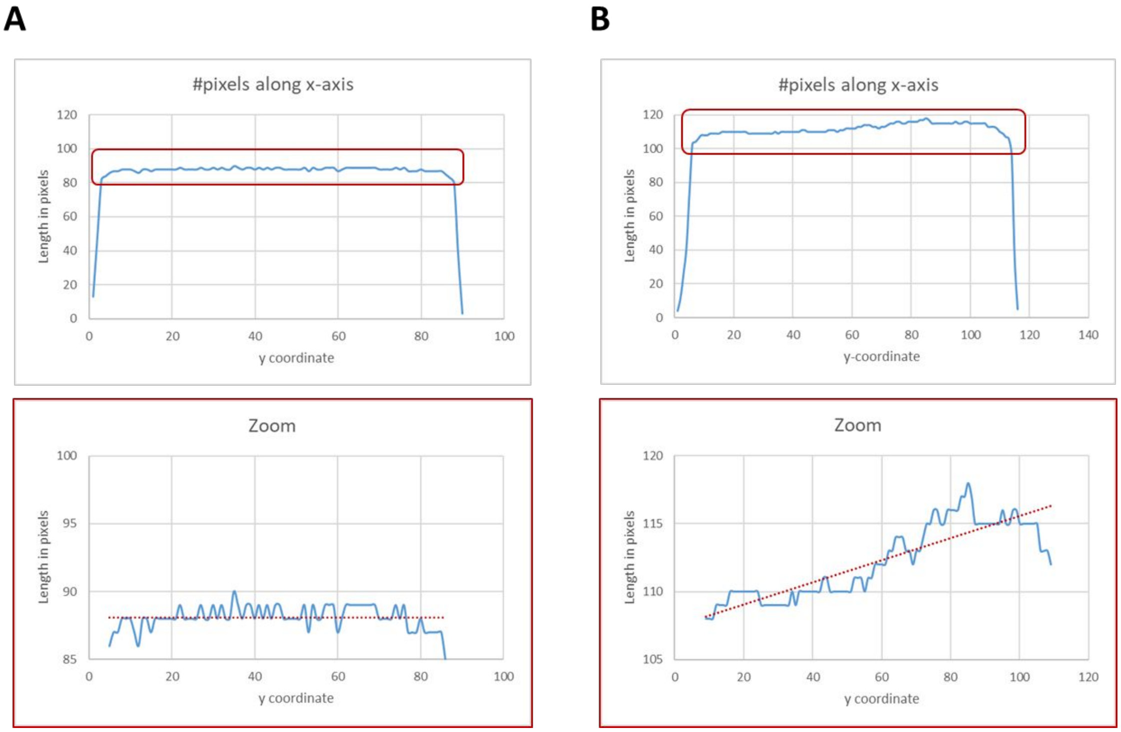

Figure S2: Number of internal pixels along *x* as a function of the pixel line for A) the isotropic square represented in figure 3 and B) the square exhibiting local distortions displayed in figure 4. Slopes of the fitted trendlines are for A) 0.0003 and for B) 0.081.

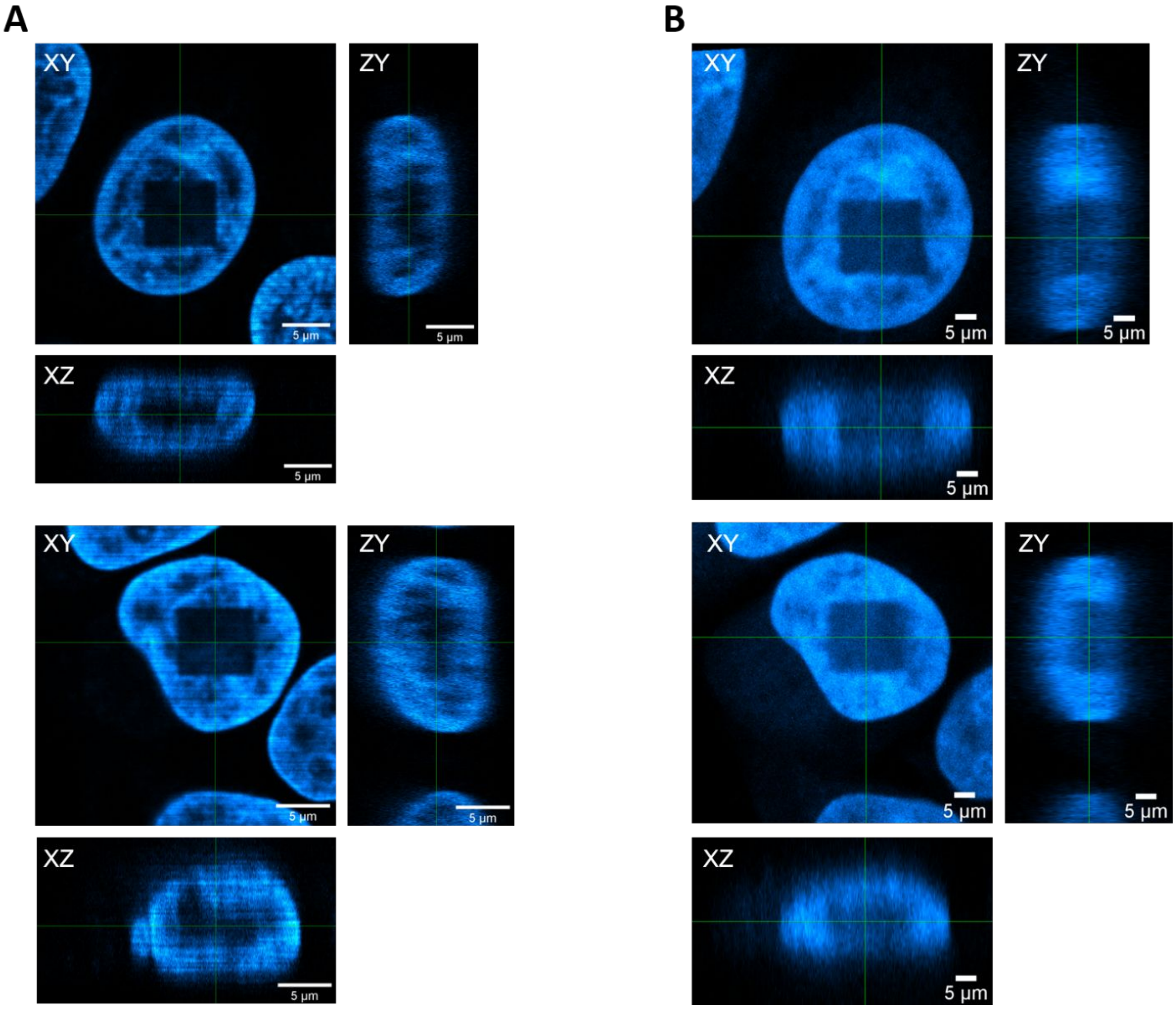

Figure S3: A) Pre- and B) post-expansion fluorescence photobleached cubes in a the nucleus of DAPI stained cels.

Table S1: Median expansion factors and respective median absolute deviations along *x*, *y* and *z* for 3 isotropically expanded photobleached cubes in three different cells. Here, isotropy along *x* and *y* was found to be concomitant with isotropy along *z*.

	**Cube 1**			**Cube 2**			**Cube 3**		
**Dimension**	**X**	**Y**	**Z**	**X**	**Y**	**Z**	**X**	**Y**	**Z**
**n**	102	105	193	62	65	123	106	116	212
**Median EF**	4.94	4.91	4.86	5.35	5.59	5.52	4.17	4.13	3.96
**MAD**	0.05	0.05	0.35	0.08	0.08	0.69	0.04	0.04	0.31

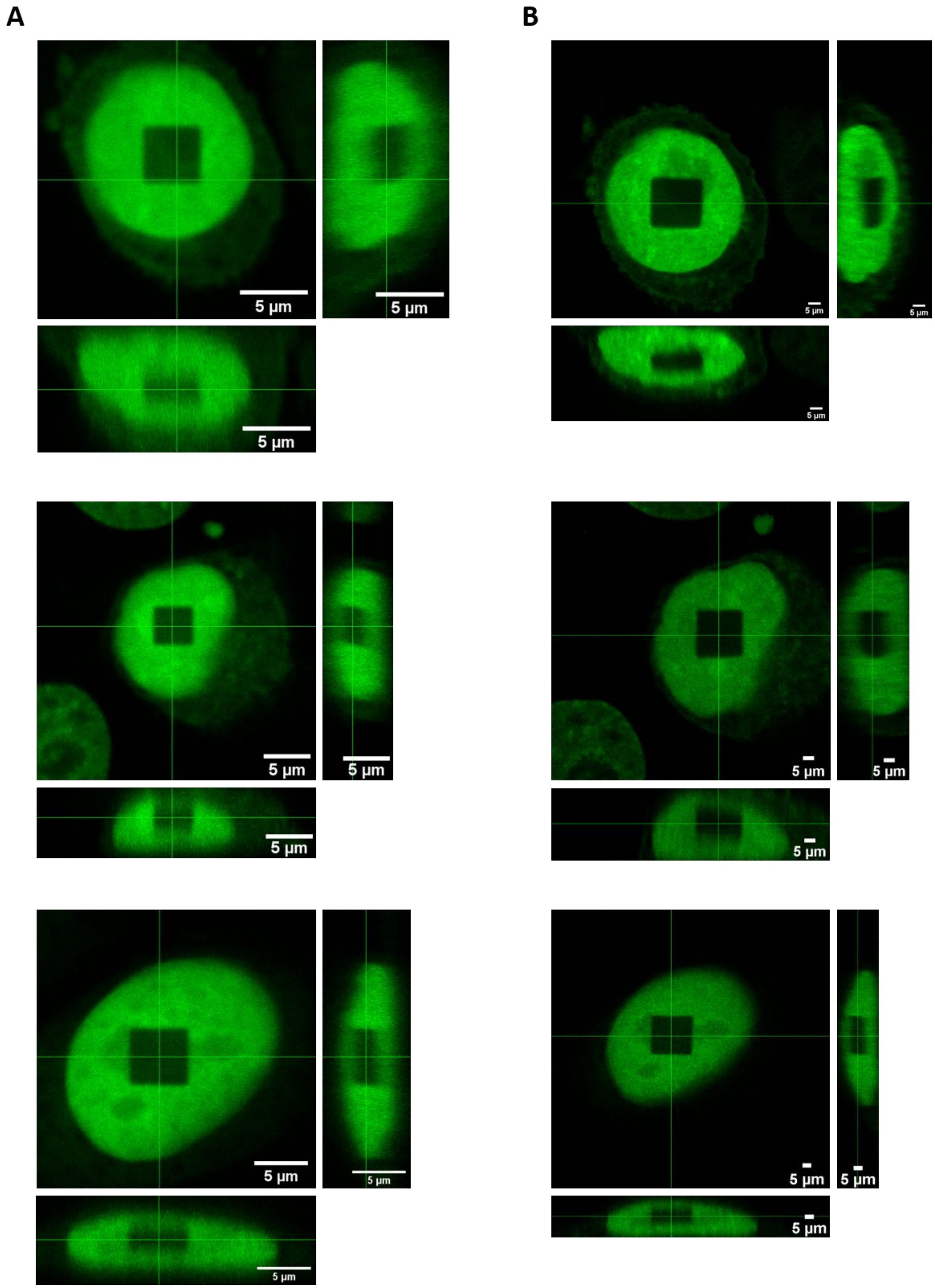

Figure S4: 3 Additional examples of isotropically expanded cubes represented in table 1. A) Pre- and B) post-expansion orthogonal views of cell 1 (upper panel), cell 2 (middle panel) and cell 3 (lower panel).

Script used in Fiji for counting pixels after the plane was selected which you want to use for downstream analysis and after post-processing of the image (see materials and methods for details):

```
// Pre-ExM
run("Analyze Particles...", "size=100-Infinity pixel display exclude clear add");
// In the case that more ROI’s are found, manually remove the ROI’s which are not representing // the square
roiManager("Select", 0);
getSelectionBounds(x, y, width, height)
makeRectangle(x, y, width, height)
run("Crop");
rename("PRE")

//Post-ExM
run("Analyze Particles...", "size=100-Infinity pixel display exclude clear add");
// In the case that more ROI’s are found, manually remove the ROI’s which are not representing // the square
roiManager("Select", 0);
getSelectionBounds(x, y, width, height)
makeRectangle(x, y, width, height)
run("Crop");
rename("POST")

//rescale images
selectWindow("PRE")
heightPRE = getHeight()
widthPRE = getWidth()
selectWindow("POST")
heightPOST = getHeight()
widthPOST = getWidth()
if (heightPOST>heightPRE) {
	run("Size...", "width=widthPRE height=heightPRE depth=1 average interpolation=Bilinear");
}
if (heightPOST<heightPRE) {
	selectWindow("PRE")
	run("Size...", "width=widthPOST height=heightPOST depth=1 average interpolation=Bilinear");
}
setAutoThreshold()
run("Convert to Mask")

//Returns the number of times the value occurs within line y pre-expansion
selectWindow("PRE")
```

```
getDimensions(width, height, channels, slices, frames)
for (i=0; i<height; i++) {
            countX=0;
   for (a=0; a<width; a++) {
      if (getPixel(a,i)==0) {
         countX++;
      }
   }
   setResult("countX", i, countX);}
```

//Returns the actual dimensions in X pre-expansion

```
getPixelSize(unit, pixelWidth, pixelHeight)
dimensionX=0
for (i = 0; i < nResults; i++) {
            dimensionX= getResult("countX", i)*pixelWidth;
            setResult("dimensionX", i, dimensionX);}
```

//Returns the number of times the value occurs within row x pre-expansion

```
getDimensions(width, height, channels, slices, frames)
for (i=0; i<width; i++) {
            countY=0;
   for (a=0; a<height; a++) {
      if (getPixel(i,a)==0) {
         countY++;
      }
   }
   setResult("countY", i, countY);}
```

//Returns the actual dimension in Y pre-expansion

```
dimensionY=0
for (i = 0; i < nResults; i++) {
            dimensionY= getResult("countY", i)*pixelHeight;
            setResult("dimensionY", i, dimensionY);}
```

//Returns the number of times the value occurs within line y post-expansion

```
selectWindow("POST")
getDimensions(width, height, channels, slices, frames)
for (i=0; i<height; i++) {
            countXpost=0;
   for (a=0; a<width; a++) {
```

```
        if (getPixel(a,i)==0) {
           countXpost++;
        }
    }
    setResult("countXpost", i, countXpost);}

//Returns the actual dimensions in X post-expansion
getPixelSize(unit, pixelWidth, pixelHeight)
dimensionXpost=0
for (i = 0; i < nResults; i++) {
            dimensionXpost= getResult("countXpost", i)*pixelWidth;
            setResult("dimensionXpost", i, dimensionXpost);}

//Returns the number of times the value occurs within row x post-expansion
getDimensions(width, height, channels, slices, frames)
for (i=0; i<width; i++) {
            countYpost=0;
    for (a=0; a<height; a++) {
        if (getPixel(i,a)==0) {
           countYpost++;
        }
    }
    setResult("countYpost", i, countYpost);}

//Returns the actual dimension in Y post-expansion
dimensionYpost=0
for (i = 0; i < nResults; i++) {
            dimensionYpost= getResult("countYpost", i)*pixelHeight;
            setResult("dimensionYpost", i, dimensionYpost);}

//calculate expansion factor X and expansion factor Y for each line
ExpansionfactorX=0
for (i = 0; i < nResults; i++) {
            ExpansionfactorX=getResult("dimensionXpost", i)/getResult("dimensionX", i);
            setResult("ExpansionfactorX", i, ExpansionfactorX);
}

ExpansionfactorY=0
for (i = 0; i < nResults; i++) {
            ExpansionfactorY=getResult("dimensionYpost", i)/getResult("dimensionY", i);
            setResult("ExpansionfactorY", i, ExpansionfactorY);
}
```
